# Offshore Exploration in the Arctic: Can Shell’s Oil-Spill Response Plans Keep Up?

**DOI:** 10.1289/ehp.120-a194

**Published:** 2012-05-01

**Authors:** Charles W. Schmidt

**Affiliations:** **Charles W. Schmidt**, MS, an award-winning science writer from Portland, ME, has written for *Discover Magazine*, *Science*, and *Nature Medicine*.


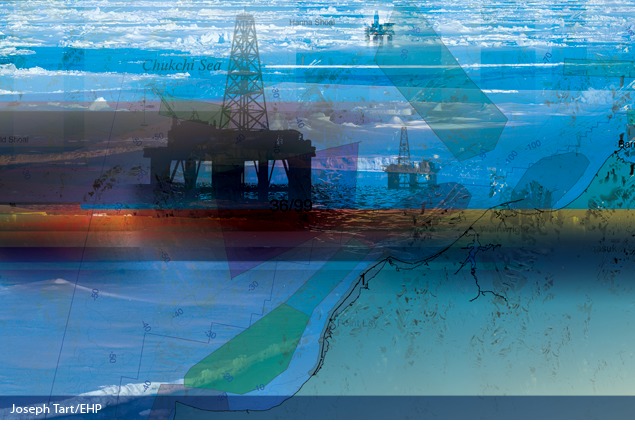


All around the world, oil and gas companies are being forced by resource declines to drill in less accessible areas, and the Arctic is their newest frontier. The geology above the Arctic Circle—that is, everything above latitude 66.56°N—holds an estimated 90 billion barrels of oil and 1,669 trillion cubic feet of natural gas, or 22% of the world’s undiscovered^^1^^ conventional resources, according to the U.S. Energy Information Administration.^^2^^ It’s thought that these resources lie predominantly under Arctic seas, which have recently become easier to reach due to significant reductions in seasonal ice cover associated with global climate change.^^3^^ Norway, Russia, Canada, Denmark, and other northern countries are in various stages of developing offshore Arctic programs, and diplomatic squabbles have broken out over territorial rights extending all the way to the North Pole.

Between 1980 and 2000 Alaska accounted for an average one-fifth of U.S. oil production.^^4^^ But with oil flowing through the Trans-Alaska Pipeline falling by more than two-thirds since a peak in 1988,^^4^^ the recent pressure to drill in the Outer Continental Shelf (OCS) off the state’s north coast has been relentless. Alaska derives at least 90% of its revenues from oil,^^5^^ so law makers in that state—supported by much of the state’s population—have pushed hard for offshore authorization. The federal government has also indicated its support, in 2011 expressing a commitment to facilitate development in the OCS region and to expedite offshore permitting in Alaska, assuming that “safety, health, and environmental standards are fully met.”^^6^^

Now the United States has taken a big step toward opening the seas off Alaska to a new round of oil and gas exploration. In a major breakthrough for the petroleum industry and loss for drilling opponents, the U.S. Department of the Interior (DOI) in February 2012 approved Shell Gulf of Mexico, Inc.’s oil-spill response plan for the Chukchi Sea, which provides habitat for polar bears, walruses, and other wildlife, and a hunting ground for Alaska Natives who still go whaling in seal-skin boats.^^7^^ Six weeks later the company’s spill-response plan for the adjoining Beaufort Sea also was approved.^8^

**Figure:**
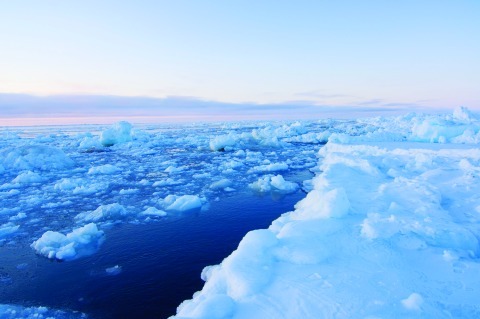
Shell must cease drilling well before the water freezes over lest a late-season blowout cause oil to accumulate under the ice through the winter. © Steven J. Kazlowski/Alamy

Shell Alaska spokesman Curtis Smith says DOI approval of the plans puts the company on track to launch exploration in the region this summer (final permits to drill must still be obtained). But many critics contend it’s not possible to drill safely in the region, given the isolation and harsh weather, and they question how well the plans will protect Arctic health in the event of a spill.

## Native Health Concerns

Access to the Alaska OCS has been blocked in recent years mainly by lawyers representing Alaska Natives, who argue that apart from its ecological consequences, offshore drilling could hurt the traditional livelihoods, health, and well-being of these local residents. The Inupiat people have hunted bowhead whales and other marine species in Arctic waters for well over 2,000 years, and half their caloric intake comes from subsistence sources of meat.^^9^^ Health studies of the native population have associated the oil industry’s expansion in the North Slope to disruption of the traditional subsistence lifestyle, contributing to rising rates of type 2 diabetes, metabolic problems from changing diets, substance abuse, suicide, and asthma.^^9^^

**Figure:**
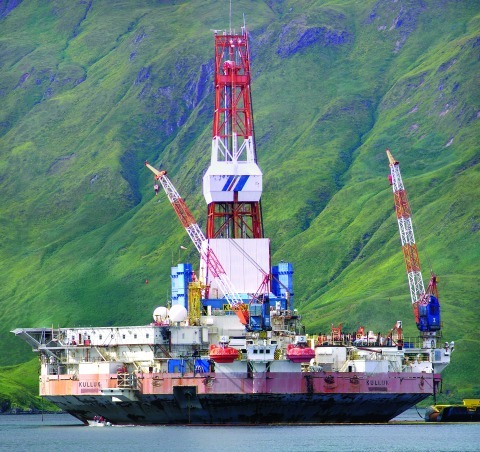
A second drill rig engaged in Beaufort Sea exploration—the Kulluk—could begin drilling a relief well in the Chukchi within a week should attempts to kill a worst-case blowout fail. © Tom Doyle

Meanwhile, during fall migration bowhead whales have been documented to travel up to 18 miles to avoid sounds they don’t like,^^10^^ potentially putting them beyond safe reach of a hunt that is crucial to the Inupiat’s cultural identity. “For every additional mile a whaler has to travel, there’s more potential for injury or a potentially catastrophic event,” says Thomas Lohman, an environmental resource specialist in the North Slope Borough^11^ Department of Wildlife Management.

The 2011 exploration season was blocked in part by Alaska Native health concerns having to do with Shell’s air permits sought from the U.S. Environmental Protection Agency (EPA).^^12^^ In that case, lawyers argued successfully that ships in the offshore drilling fleet—particularly icebreakers—would emit excessive amounts of nitrogen oxides, respiratory irritants linked with heart disease. (That issue has been resolved by Shell and the EPA, and final air permits for the *Kulluk* and *Noble Discoverer* rigs were issued in October 2011 and February 2012, respectively.^^13^^^^,^^^^14^^) According to Smith, Shell will deploy best-available pollution-control technology on its drill rigs, and all its aircraft in the region will use ultra-low-sulfur diesel fuel, which substantially cuts emissions of nitrogen oxides and particulate matter.^15^

**Figure:**
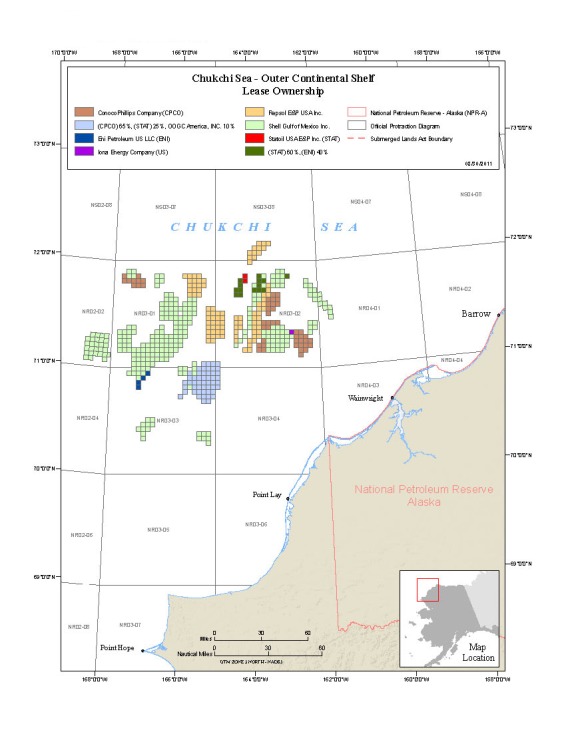
Adapted from maps by Shell7 and BOEMRE.^36^

Lohman concedes that Shell has worked hard and spent a lot of money to address local concerns. “There’s more of a partnership and dialogue now with the company than there used to be,” he says. “But what comes up again and again when you talk to local communities is the oil-spill scenario. People worry what will happen to their food supply if things really get out of control.”

## Exploring the OCS

Scientists believe the geology underlying the Chukchi and Beaufort seas contains a short-term energy bonanza: 23.6 billion barrels of oil and 104.4 trillion cubic feet of natural gas, according to recent government estimates.^^16^^ (By comparison, the United States will consume an estimated 7.3 billion barrels of oil in 2012.^^17^^) Exploration involves drilling just a few wells to confirm that the predicted size and dimensions of the resource—estimated with seismic technology—are correct. Shell’s current plans for the Chukchi Sea are to drill up to six wells over the next two years in what’s known as the Burger Prospect, about 70 miles offshore in roughly 140 feet of water. The company also plans to drill four wells over two years in the Beaufort Sea at a shallower depth, pending further DOI review.

Unlike development and production (the year-round process of constructing the necessary facilities and extracting oil and gas for delivery to market), exploration will happen only in summer, when the seas are mostly ice-free.^^18^^ Shell’s oil-spill response plans were developed specifically for conditions expected from July 15 to the end of October, but the vessels and equipment are designed to work past this period if needed, with contingencies for prolonged cleanup through late fall, Smith says.

With more than $4 billion invested in offshore Arctic infrastructure, research, and leases,^^19^^ Shell has been seeking approval for OCS exploration every summer since 2006. But given that northern seas offer some of the most challenging drilling conditions on Earth^^20^^ in an area that’s also home to an array of vulnerable and iconic species of wildlife, the company’s plans have drawn heavy scrutiny from federal agencies and a wide range of passionate stakeholders. Drilling opponents and some oil-spill veterans assert that the extreme cold, storms, high waves, winds, darkness, and fog that occur routinely in the region could challenge spill cleanup, particularly in the event of a late-fall blowout, when ice begins to gather.^^21^^ What’s more, OCS waters are exceedingly remote—roads, airports, port facilities, housing, and other infrastructure needed to support a large-scale spill response are few and far between.^^16^^

Smith responds that Shell’s oil-spill response plans are the most far-reaching developed by the company yet for any of its global operations. As required by the DOI in the wake of the 2010 BP *Deepwater Horizon* blowout in the Gulf of Mexico, the plans describes worst-case discharges of 25,000 barrels of oil per day in the Chukchi^^7^^ and 16,000 barrels per day in the Beaufort.^^8^^ These figures, which Smith says reflect the likely pressures and other characteristics of the respective reservoirs, are considered more realistic than the 5,500 barrels per day that was considered in earlier plans. Also in response the *Deepwater Horizon* disaster, the DOI requires that Shell have access to a “capping stack” to stanch subsea oil flows in the event that other shutoff systems fail (the BP blowout was eventually contained by such a device) in addition to capabilities to capture and collect oil from the capping stack, and ready access to a rig that could kill a blowout by drilling a relief well.

## Differences from Deepwater Horizon

Multiple factors play in Shell’s favor in the OCS, says Peter Velez, Shell’s global emergency response manager. For instance, unlike BP’s ill-fated Macondo Prospect, site of the *Deepwater Horizon* blowout, which occurred 5,000 feet underwater, Shell’s proposed sites off Alaska are in less than 150 feet of water, making them more accessible to divers and remotely operated vehicles deployed during spill response, he says. Moreover, well pressures at the proposed sites aren’t expected to exceed 3,000–4,000 psi, compared with the Macondo well’s pressure of almost 15,000 psi, making a blowout less likely to occur, Velez says.

All the same, to avoid the risk of a late-season blowout, the DOI’s Bureau of Ocean Energy Management (BOEM)^^22^^ required that Shell cease drilling into known hydrocarbon zones in the Chukchi Sea by September 24, just over a month before ice is expected to begin covering the proposed sites.^^23^^ (Drilling in the Beaufort Sea, according to the BOEM, must also be suspended by August 25 to avoid interfering with whale hunts by the Nuiqsut and Kaktovik people but may resume after the hunters have reached their quota.^^24^^) Commenting on the Chukchi plan upon its approval, James A. Watson, director of the DOI Bureau of Safety and Environmental Enforcement, said, “After an exhaustive review, we have confidence that Shell’s plan includes the necessary equipment and personnel prestaging, training, logistics, and communication to act quickly and mount an effective response should a spill occur.”^^25^^

If Shell’s summer exploration confirms that the oil resource is economically viable, then not just Shell but also other companies will begin planning in earnest for year-round development, suggesting that at some point in the future the OCS may be populated by numerous drill rigs operating simultaneously. Shell’s plans describe spill responses under “varying ice conditions,” but importantly, they don’t address the near-total ice cover anticipated during midwinter development, which Smith says is at least a decade away.

How to address oil accumulating under completely frozen seas—a nightmare scenario, many scientists say—remains somewhat of an open question. “My concern is that year-round development will require adequate capacity to respond to spills in icy, dark conditions. And so far, I haven’t seen a demonstration of that capacity anywhere,” says Roger Rufe, a retired vice admiral in the U.S. Coast Guard, who served as district commander in Alaska from 1992 to 1995. Commenting on spill cleanup during midwinter, Smith responds, “We’re looking at this now, but we haven’t made any fixed decisions about what we’re going to do. What I can say is that whatever plan we produce will be completely transparent, and it will undergo the same scrutiny as the response plan for summer exploration that we have now.”

During the *Deepwater Horizon* disaster, thousands of response workers and hundreds of air- and seacraft were deployed from the Gulf’s highly developed coastline. Moreover, the response was coordinated by a consortium of oil companies, each contributing resources and manpower to the cleanup effort. But in the Alaskan OCS, Shell has to rely on its own, much more limited resources, which in the Chukchi include the drill rig itself (the *Noble Discoverer*, making its way to the OCS from New Zealand at press time), an oil-spill response vessel (the *Nanuq*, which carries smaller workboats, booms, storage for recovered oil, and a dispersant system), a pair of large barges that carry oil skimmers and storage capacity for recovered oil, an Arctic storage tanker (the *Affinity*, with 513,000 barrels of storage space, to be positioned within 240 nautical miles of the *Noble Discoverer*), and an assortment of shoreline protection equipment, landing craft, and other work boats. A second drill rig engaged in Beaufort Sea exploration—the *Kulluk*—could begin drilling a relief well in the Chukchi within a week should attempts to kill a worst-case blowout fail, Velez says.

## Blowout Prevention

Blowouts start with what’s known as a kick, or a blast of pressurized oil and gas that suddenly bursts up the wellbore and into the drill pipe. A five-story blowout preventer (BOP) built over the wellhead should activate “blind shear rams” that cut the drill pipe, seal the well bore, and kill the well.^^7^^^^,^^^^8^^

But that doesn’t always happen: in the Gulf of Mexico BP fatefully relied on a BOP with just one blind shear ram, which failed to engage, leaving nothing to stop a full-scale blowout on 20 April 2010. Eleven workers lost their lives when the rig exploded, and the rogue well released 4.9 billion barrels (205.8 million gallons) of oil into the sea before it was killed nearly three months later.^^26^^ For backup in the Arctic, Shell’s BOP will have not one but two blind shear rams, and if they fail, the 100-ton capping stack ideally will land on the dysfunctional BOP and either seal the well completely or divert the gushing oil to a container ship.^^7^^^^,^^^^8^^

As of this writing, Shell’s Arctic capping stack is still under construction; the company plans to test it in the Pacific Ocean off Seattle, much to the dismay of environmental activists who say it should be tested in the Arctic OCS. “Testing in Washington could answer a few logistical questions about how it would be deployed,” says Layla Hughes, an attorney and senior program officer with the World Wildlife Fund’s Arctic team in Juneau. “But it doesn’t give a sense of the real-world limitations of using the capping stack in the Arctic in bad weather.”

Shell claims its oil-spill response will kick into gear within an hour of a blowout. The company divides its worst-case scenario into icy and non-icy conditions, which is important because ice cover dictates how and whether the company can deploy booms and skimmers for so-called mechanical oil-spill recovery.

Booms float on the surface and act as barriers to prevent oil from spreading. They can also be used to gather oil into thick U-shaped pools, so it can be burned on the surface (“*in-situ* burning”) or pulled out of the water by skimmers for storage. Shell plans to use fire-resistant booms and two types of skimmers: weir skimmers, which suck oil and water off the surface like a vacuum cleaner (the oil is separated later), and brush skimmers, which spin in the water and trap oil on rotating fibers.

Booms don’t work when sea-surface ice cover exceeds 30%, claims Stein Erik Sørstrøm, research manager with SINTEF (Stiftelsen for Industriell og Teknisk Forskning, or Foundation for Scientific and Industrial Research), an independent research organization based in Trondheim, Norway. But he also points out that, at a high enough percentage of cover, ice can itself act as a sort of boom. (Sørstrøm directed SINTEF’s Joint Industry Project Oil in Ice program, a collaboration of six multinational oil companies, including Shell, that investigated oil-spill response scenarios in the laboratory and then in Arctic seas halfway between Norway and the North Pole.)^^27^^

According to SINTEF’s research findings, booms and skimmers work best in calm, ice-free conditions. But at wind speeds over 22 knots, oil starts sloshing over the booms, while ice buildup clogs skimmers and reduces their efficiency.^^7^^ Shell’s Chukchi plan asserts that from July to September, wind speeds average 10–13 knots in a prevailing northeast direction that carries oil away from shore.^^7^^

## In-Situ Burning

When heavy weather gets in the way of mechanical recovery, Shell’s plans shift chiefly to nonmechanical methods: namely, *in-situ* burning and the use of chemical dispersants. Both of these methods are contingent on approval from the Coast Guard, which by federal law is in charge of the response (although it does not actually perform the cleanup).

Under optimal conditions, *in-situ* burning can remove 85–95% of the oil, but that depends on a number of factors.^^28^^ In particular, the oil needs to be fresh, meaning that its combustible volatile fractions haven’t yet been lost to evaporation. Oil weathers over time, leaving less combustible fractions behind. It also mixes with water, making it less able to ignite—oily emulsifications won’t burn if they contain more than 25% water.^^29^^ High winds can also make it difficult for crews to ignite floating oil. A 2011 report by the Ottawa, Ontario–based firm S.L. Ross Environmental Research, Ltd., which consults to both industry and the Canadian government, claims that *in-situ* burning isn’t possible in open water with waves higher than 1.5 meters or at wind speeds of more than 20 knots,^^29^^ conditions that occur routinely in the OCS.

**Figure:**
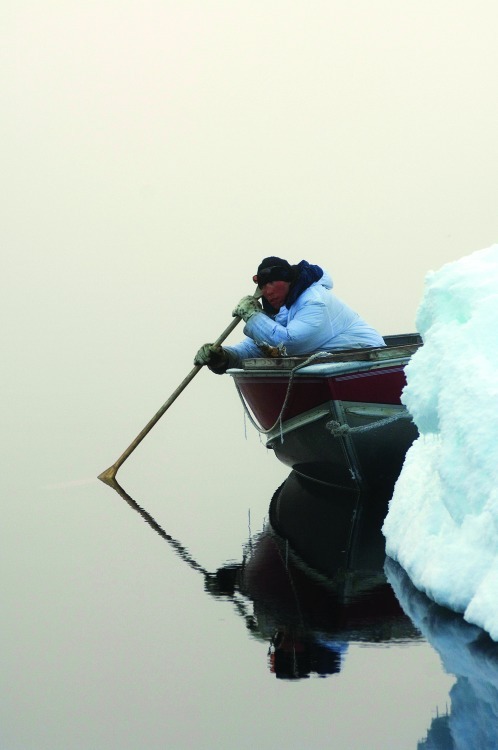
An Inupiaq hunter uses an oar to listen for passing whales in the Chukchi Sea. Environmental resource specialist Thomas Lohman acknowledges that Shell has worked hard to address local concerns, but adds, “What comes up again and again when you talk to local communities is the oil-spill scenario. People worry what will happen to their food supply if things really get out of control.” © Steven J. Kazlowski/Alamy

On the other hand, cold Arctic temperatures limit evaporation, and this slows weathering, according to Sørstrøm. And although it’s not expected during summer, ice coverage of up to 80% also favors *in-situ* burning, because it dampens waves and concentrates oil into dense floating pockets. At higher coverage, ice blocks access to the oil, but Shell has contingencies for that: its plans call for “ice management,” or using ships to hold the ice at bay or break it into smaller pieces. When ice coverage becomes so extensive that cleanup isn’t no longer possible, the plans call for halting the response until spring.

“We’ve done experiments showing that oil encapsulated in ice will remain [unweathered] through the winter,” says Steve Potter, vice president and director of S.L. Ross. “So when the ice melts, the oil will appear on the surface, and you can deal with it then.”

Shell’s plans claim that oil encapsulated in ice won’t come in contact with wildlife. But Richard Steiner, a conservation biologist with Anchorage consulting service Oasis Earth, counters that there’s a lot of microbial life in the interface where floating ice meets the sea, and that this layer also is the site of a great deal of “primary production,” or the conversion of aquatic carbon dioxide into life-sustaining organic molecules. Moreover, the floating sheet of ice that makes up the sea surface will travel, he says, and spread the oil when it melts.

## Chemical Dispersants

Chemical dispersants make up the third leg of Shell’s spill response plans. Shell intends to rely mainly on a product used during the *Deepwater Horizon* response: Corexit^→^ 9500. This combination of petroleum distillates, surfactants, and stabilizers allows oil to mix more easily into water, where it can be degraded by marine bacteria. During the *Deepwater Horizon* blowout, BP applied Corexit directly at the gushing wellhead on the seafloor; Shell intends to apply it mostly from the air, specifically from a Lockheed C-130 Hercules military plane or from a vessel.^^7^^^^,^^^^8^^ But chemical dispersants aren’t preapproved for use in Alaska and would be considered only when other response measures aren’t working, according to the Alaska Regional Response Team, which is charged with developing contingency plans to coordinate multiagency disaster responses.^^30^^

Meanwhile, Ken Trudel, a senior environmental scientist with S.L. Ross, says investigators don’t have much information about how dispersants might work under real-world Arctic conditions. SINTEF conducted the first significant field tests, showing that Corexit applied from floating vessels onto both fresh and week-old oil achieved dispersing efficiencies of greater than 90% (aerial spraying wasn’t evaluated).^^31^^ But targeting oil between ice floes was challenging, that study found. “When you’ve got oil on open water, all you have to do is spray it,” Trudel says. “But when the oil’s between ice floes, you have to find it, spray the dispersant right on to the oil, and then it’s harder to find out if it’s working because you can’t see it as well.”

According to Trudel, dispersants work best on fresh oil in choppy seas that mix the chemicals into the water. Shell plans to create turbulence with its vessels’ propellers if the seas are icy or flat, and to use dispersants only until several days after the blowout is contained.

Both dispersant use and *in-situ* burning have ecological consequences. Some studies suggest that chemically dispersed oil can be more toxic to marine life than undispersed oil,^^32^^ although this remains an open area of research. And according to a November 2010 report commissioned by the Pew Environment Group’s U.S. Arctic program, charred residues left over from *in-situ* burning aren’t as toxic as the original oil, but they’re hardly benign.^^32^^ Shell’s plans call for manually removing as much of this residue as possible^^7^^^^,^^^^8^^—a laborious process involving strainers, nets, sorbents, and skimmers—but whatever sinks to the bottom can contaminate benthic ecosystems.^^32^^

Although Shell’s plans describe detailed contingencies for cleanup in ice, Smith emphasizes that summer exploration will occur in open water and nearly perpetual daylight. During this time, the main limiting factors will be wind, waves, and fog (which reduces visibility for aerial dispersant application), each of which becomes increasingly problematic as the season progresses.^^30^^

Weather conditions in the Beaufort Sea could make it impossible to mount any oil-spill response whatsoever 22% of the time in July, 41% of the time in August, and 56% of the time in September.^^29^^ That increase over time results mainly from daylight losses that become more pronounced as fall draws near—daylight starts to lessen in September, and darkness rules the region from November through mid-February.^^7^^ Potter says aerial dispersant application isn’t advisable in darkness. “You could spray at night, but you would probably waste a lot of dispersant,” he explains. “Without visual sighting from small planes and communication with the application aircraft, it’s hard to target the slick.” (He adds that this logistical problem “seems solvable, technically, and is an area of ongoing research.”)

## Ready for Prime Time?

The question remains, however, whether these or any other oil-spill response plans will fulfill their purpose when the time comes. A report issued by the U.S. Government Accountability Office (GAO) as this article went to press notes that, although the oil industry has improved its capability to respond to subsea blowouts in the Gulf of Mexico, these improvements may not be enough to overcome all the environmental and logistical challenges associated with drilling in the Arctic. Moreover, the report indicates that although the DOI has instituted more rigorous requirements for companies’ oil-spill response plans since the *Deepwater Horizon* disaster, the department has not documented a consistent procedure for evaluating these plans and ensuring that companies can actually carry them out.^^33^^

The GAO report echoed a 2011 report commissioned by DOI Secretary Kenneth Salazar to assess the state of the science on offshore Arctic drilling, which revealed significant unknowns about how oil and gas activities might affect local ecology. That report also raised major questions about whether oil companies could respond adequately to a major spill in the region.^^34^^

As Shell finally moves toward summer exploration, its work in the OCS is expected to attract tremendous public attention. In February 2012 the *Noble Discoverer* drill rig was boarded by protesters in New Zealand, notably by actress Lucy Lawless (who, ironically, appeared as a gas station attendant in a Shell commercial 20 years ago^^35^^). And in March, Greenpeace activists in Finland raided two Shell icebreakers bound for the Chukchi Sea. Smith expects that protesters will try to raid Shell’s ships in the OCS this summer.

But it’s highly improbable that Shell will spill any oil, at least in significant amounts, this year. Drilling just a few wells under mainly sunny skies and in more or less ice-free seas doesn’t constitute the real threat to the Alaskan OCS. Exploration merely sets the stage for the much greater threat that comes later, at the point of development. Shell’s plan may have satisfied the DOI’s requirements for a limited venture this summer. But questions about how the oil industry will protect this fragile ecosystem and the people who live there if development begins full-tilt remain unanswered.
